# Online education and its relation to hearing status among higher-secondary students in Bangladesh: A cross-sectional survey

**DOI:** 10.1371/journal.pone.0342668

**Published:** 2026-02-13

**Authors:** Syeda Tasnim Tabassum Hridi, Mohammad Azmain Iktidar, Kazi Sudipta Kabir, Punam Ghosh, Arrafi Tamjid, Abdullah Al Zaber, Rifat Tasnim Babu, Maliha Mehzabeen, Aysharja Das Gupta, Sreshtha Chowdhury, Simanta Roy

**Affiliations:** 1 North East Medical College, Sylhet, Bangladesh; 2 Directorate General of Health Services, Dhaka, Bangladesh; 3 School of Research, Chattogram, Bangladesh; 4 PMK Hospital and Diagnostic Center, Dhaka, Bangladesh; 5 Chattogram Medical College Hospital, Chattogram, Bangladesh; 6 North South University, Dhaka, Bangladesh; 7 International Centre for Diarrhoeal Disease Research, Bangladesh; 8 BGC Trust Medical College and Hospital, Chattogram, Bangladesh; Shenyang Jianzhu University, CHINA

## Abstract

**Background:**

Online education gained its popularity in the education system during the COVID-19 pandemic lockdown. The online platform, including social media, was institutionalized globally for the purpose of tutoring to keep the education process ongoing under feasible circumstances. However, the post-pandemic continuation of online education and prolonged usage of electronic devices imposed a greater risk of health issues related to sensory impairment. Our study aimed to determine the impact of online education on students’ hearing status and its associated factors.

**Methods:**

A cross-sectional study was conducted among 1030 students of 11th grade and above who were undergoing online education in Dhaka and Chattogram. Data were collected through the online administration of a structured questionnaire containing questions on sociodemographic status, family history of diseases, personal history of diseases, information related to screentime exposure, and SSQ-12 (Speech, Spatial, and Qualities of Hearing −12) scale. Descriptive statistics, Pearson’s chi-square test, two independent sample t-tests, and multiple linear regression analysis were employed to obtain the results.

**Result:**

The mean SSQ score of the study participants was 7.74 ± 1.37. In bivariate analysis, gender, family income, family history of diseases (e.g., obesity, headache, hearing problem), personal history of diseases (e.g., obesity, insomnia), device type (mobile/tablet, computer), average daily screen time with sound, and break pattern during online learning were significantly (p < 0.05 for all) associated with hearing status. In multivariate analysis, being female (coefficient −0.293, p = 0.001), using mobile/tablet (coefficient −0.836, p = 0.001), and continuous screen use (coefficient −0.348, p = 0.003) were significantly associated with poor hearing status.

**Conclusion:**

This current study indicates the detrimental effect of online education on the hearing of young students in Bangladesh. Future studies should explore the long-term hearing effects of online education and guide the policy makers towards necessary preventive approaches.

## Introduction

Online education, or virtual learning as an innovative approach, offers the flexibility to access educational resources such as learning materials and lectures with the power of the internet and digital technologies [1]. By breaking down barriers and promoting inclusivity, online education has become a powerful tool in democratizing access to knowledge and empowering learners worldwide [2].

The COVID-19 pandemic marked a watershed moment in the adoption of online education. Widespread lockdowns necessitated an abrupt shift to virtual learning, propelling its adoption to unprecedented levels [3]. This shift affected individuals worldwide, from school-level students to university scholars, who increasingly relied on mobile phones or laptops for virtual learning. For instance, a cross-sectional study conducted in Bangladesh revealed that students engaged in online learning for an average of two to six hours daily during the pandemic. Such extended distance learning period requires prolonged use of audio devices like headphones and speakers, raising concerns about potential auditory health risks [4]. Even post-pandemic, online education has retained its relevance due to its convenience and adaptability while raising concerns about potential health risks associated with prolonged online learning [5].

For online education, students relied on headphones or speakers, varying in sound quality, and participated in diverse learning environments with sub-optimal acoustic conditions [6]. A recent cross-sectional study by Dessai et al. (2023) reported a range of hearing-related symptoms such as ear pain and tinnitus of varying severity among students participating in online classes, suggesting a potential risk to auditory health associated with prolonged engagement in online education [7]. Another nationwide survey in South Korea found that one in five adolescents using earphones for at least 80 minutes daily exhibited signs of hearing loss [8]. Similarly, another cross-sectional study among adults in Andra Pradesh, India, revealed that using headphones for diverse purposes, such as leisure, education, service, music, and gaming, resulted in subclinical hearing loss [9]. In addition, using earphones in a noisy environment contributed to higher hearing impairment among adolescents [7].

In Bangladesh, where access to quiet study environments may be limited, students are often exposed to suboptimal acoustic conditions while engaging in online education. This exposure compounded by varying sound quality and prolonged usage of headphones, poses a potential risk to auditory health [5,4]. Since gadgets for online education and leisure activities have witnessed exponential growth, the risk of auditory health problems has become a pressing concern. Although age, heredity, gender, and ethnicity have been identified as factors contributing to noise-induced hearing loss [10], there remains a dearth of comprehensive data regarding the specific impact of online education on hearing. Therefore, this study seeks to address this gap by investigating the effects of online education on the hearing status of Bangladeshi students. By doing so, it aims to provide valuable insights into the auditory health risks associated with virtual learning and to underscore the importance of preventive measures in mitigating these risks.

## Methods

### Study design, sites, and participants

A cross-sectional survey was conducted between 25 May 2022 and 31 July 2023 to assess the impact of online education on the hearing status of the students studying in 11th grade and above. The study focused on students from 11th grade upward because students below that level in Bangladesh had limited access to digital gadgets and were mainly taught through government-run television programs. Preselected educational institutes, including tuition homes or academies located in Dhaka and Chattogram, were chosen to obtain data that considered the accessibility of students from diverse educational backgrounds. For the students who were 18 years and above, the informed consent was directly obtained from them. However, for participants below 18 years, informed consent was obtained from their parents. The consent form contained all the detailed information concerning the study and the probable outcome of participation.

### Sampling and selection criteria

A total of 1030 students were enrolled in the study through convenience sampling. The aforementioned sampling strategy was applied considering the limitation of resources. Even though the sampling technique lacks randomization, the large sample size (n = 1030) helped to overcome this limitation by reducing the error margin, leading to more precise results. The following inclusion criteria were considered before selecting the study participants: (1) students who provided voluntary consent for participation, (2) students who were citizens of Bangladesh, and (3) students who received online education during the period of COVID-19 lockdown and after. We excluded students under the following criteria: (1) foreign students studying in Bangladesh, (2) students with previous histories of hearing impairment, and (3) students who were engaged with online education before COVID-19 lockdown.

### Data collection techniques

A structured questionnaire was prepared for online administration through Google Forms. The students were asked to answer the questions after providing their consent electronically for participation. The questionnaire (S1 File) included information on sociodemographic status (age, gender, educational qualification, income), family history of diseases, personal history of diseases, information related to screentime exposure (daily hours spent on electronic devices for study and entertainment, type of device used, breaks taken during use, duration of online education in months), headphone (any form of personal audio devices) use and SSQ-12 scale. Mandatory items were highlighted with a red asterisk, and the relevant non-response option was also incorporated. Respondents could review their answers through the back button and change their responses if necessary. The survey was never displayed a second time once the user had filled it in to prevent duplicate entries. Of the 1050 eligible participants who agreed to participate, 1030 participants completed the entire questionnaire (completion rate: 98.09%); incomplete questionnaires were excluded from the analysis.

### Hearing assessment

The primary outcome of the study was the hearing status of the students, which was measured via Speech, Spatial and Qualities of Hearing Scale Questionnaire (SSQ-12) [11]. The SSQ-12 scale is widely validated [11–14] and a practiced tool [15,16] for assessing hearing status. We selected the SSQ-12 because it is a brief, widely validated instrument that captures speech, spatial, and sound-quality experiences relevant to everyday listening demands, with strong applicability across populations and languages [11–14,16]. The scale included 12 items under 3 sub-scales: speech scale (items 1, 2, 3, 4, 5), spatial scale (items 6, 7, 8), and qualities of hearing scale (items 9. 10, 11, 12). For each item in each sub-scale, there is a score that runs from 0 to 10. The students needed to identify with the score that resembles their own experiences of the described situation in the item and select that score. The score provided a quantitative measure of hearing disability in each domain. The poorer the hearing experience, the lower the average score.

### Statistical analysis

The obtained data were analyzed using Stata (v.17.0; StataCorp, College Station, TX, USA) statistical software. A histogram, standard Q-Q plot, and the Kolmogorov-Smirnov test were used to check for normality in continuous data. The categorical variables were summarized using frequencies and corresponding percentages. The arithmetic mean was used for quantitative data as a measure of center, and standard deviation was used as a measure of dispersion. Associations between dependent and independent variables were assessed using Pearson’s chi-square test and two independent sample t-test. A linear regression model was used to determine the factors associated with hearing status. The lowest values of the Akaike Information Criterion (AIC) and the Bayesian Information Criterion (BIC) were considered while considering the model selection. The variance inflation factor (VIF) was used to measure the presence of multicollinearity (VIF < 5 for all). A p-value of <0.05 was considered statistically significant.

### Ethics

The study proposal was reviewed and approved by the institutional review board of North South University (approval no: 2022/OR-NSU/IRB/0403). All the study participants provided written informed consent. The objective, risks, and benefits of the research were well explained to all the study participants before participation. The anonymity of the participants was maintained, and they were allowed to withdraw from the study at any time during the study period. Wherever feasible, the 1964 Declaration of Helsinki and later modifications and comparable ethical standards were followed. Data collection was voluntary, and no incentives were offered to participants. All the data were handled with confidentiality and were not disclosed anywhere. All the reporting was done according to the Checklist for Reporting Results of Internet E-Surveys (CHERRIES) guidelines [17]

## Results

A total of 1030 students participated in the study. Their background information is presented in Table 1. The mean age of the participants was 18.62 ± 3.04 years, and the majority of them were female (60.58%). Two-thirds of the students were studying in 12th grade, and one-third of the students had their family’s monthly income ranging between thirty thousand and sixty thousand BDT ($300–600). Eye problems were reported as a major concern among the students (68.74%). Most of the students (60.78%) were continuing online education for more than 12 months, and mobile/tablets were reported as the most used electronic device for both education (94.56%) and entertainment (92.62%). The majority of the students had a screen time of 2–6 hours (66.67%) for online education and less than 2 hours (43.69%) for entertainment. However, shorter break time was noted in the majority (46.21%) of the students during online education, and most of the participants (64.27%) used headphones while using their digital devices.

**Table 1:** Background information of the study participants (N = 1030).

Variables	Frequency	Percentage
**Age**		
<18 years	478	46.44
≥18 years	552	53.56
**Gender**		
Male	406	39.42
Female	624	60.58
**Education**		
11^th^ grade	24	2.33
12^th^ grade	664	66.8
Undergraduate	258	25.05
Graduate and post-graduate	84	8.16
**Family income (in BDT)**		
<10,000	96	9.32
10,000–30,000	312	30.29
30,000–60,000	384	37.28
60,000–1,00,000	174	16.89
>1,00,000	64	6.21
Family history of diseases*		
Obesity	234	22.72
Headache	538	52.23
Eye Problem	708	68.74
Insomnia	346	33.59
Hearing Problem	88	8.54
Personal history of diseases*		
Obesity	134	13.01
Headache	448	43.5
Eye Problem	708	68.74
Insomnia	152	14.76
**Headphone use**		
No	368	35.73
Yes	662	64.27
**Online education-related use**		
**Duration (months)**		
1 to 3 months	28	2.72
3 to 6 months	74	7.18
6 to 12 months	302	29.32
>12 months	626	60.78
**Used mobile/ tablet**		
No	56	5.44
Yes	974	94.56
**Used computer**		
No	666	64.66
Yes	364	35.34
**Used TV**		
No	1,008	97.86
Yes	22	2.14
**Screentime with sound/day**		
<2 hours	130	12.67
2-6 hours	684	66.67
6-12 hours	174	16.96
>12 hours	38	3.7
**Break pattern**		
Use with enough break (15 min break after every 2 hours of use)	282	27.38
Use with a small break (10 min break after every 2 hours of use)	476	46.21
Continuous use (4 hours without break)	272	26.41
**Online entertainment related use**		
**Used mobile/ tablet**		
No	76	7.38
Yes	954	92.62
**Used computer**		
No	754	73.2
Yes	276	26.8
**Used TV**		
No	748	72.62
Yes	282	27.38
**Screentime with sound/day**		
<2 hours	450	43.69
2-6 hours	432	41.94
6-12 hours	118	11.46
>12 hours	30	2.91
**Break pattern**		
Use with enough break (15 min break after every 2 hours of use)	454	44.08
Use with a small break (10 min break after every 2 hours of use)	376	36.5
Continuous use (4 hours without break)	200	19.42

* Multiple responses

BDT, Bangladeshi Taka (1 USD = 120 BDT)

Table 2 presents the bivariate relationship between the participant and device use characteristics and mean SSQ score among the study population. Male participants had a significantly higher SSQ score compared to the female counterparts (7.93 vs 7.61, p < 0.001). Participants with family history of obesity (p < 0.001), headache (p < 0.001), insomnia (p < 0.001), hearing problem (p = 0.021), and personal history of obesity(p = 0.006), insomnia (p < 0.001), mobile use for online education (p < 0.001), continued use without break during online education (p = 0.013), and entertainment (p = 0.006) had significantly lower SSQ scores. Computer usage for online education (p < 0.001) was associated with higher SSQ score.

**Table 2:** Bivariate relationship between SSQ score and participant characteristics.

Variable name	SSQ score		
**Mean**	**SD**	**p-value**
**Age (years)**			0.3592
<18	7.77	1.29	
≥18	7.68	1.41	
**Gender**			<**0.001**
Male	7.93	1.21	
Female	7.61	1.45	
**Education**			0.2054
11^th^ grade	7.44	1.31	
12^th^ grade	7.72	1.37	
Undergraduate	7.73	1.34	
Graduate and post-graduate	8.01	1.47	
**Income (in BDT)**			<**0.001**
<10,000	7.61	1.68	
10,000–30,000	7.60	1.40	
30,000–60,000	7.77	1.38	
60,000–1,00,000	7.92	1.11	
>1,00,000	7.95	1.22	
**Family history of diseases**			
**Obesity**			<**0.001**
No	7.83	1.35	
Yes	7.42	1.40	
**Headache**			<**0.001**
No	8.00	1.22	
Yes	7.50	1.45	
**Insomnia**			<**0.001**
No	7.87	1.34	
Yes	7.47	1.53	
**Hearing Problem**			**0.02**
No	7.77	1.35	
Yes	7.42	1.53	
**Personal history of diseases**			
**Obesity**			**0.006**
No	7.78	1.34	
Yes	7.44	1.54	
**Headache**			0.07
No	7.81	1.28	
Yes	7.65	1.48	
**Insomnia**			<**0.001**
No	7.82	1.33	
Yes	7.25	1.50	
**Headphone use**			0.28
No	7.80	1.41	
Yes	7.70	1.35	
**Online Education-Related Variables**			
**Duration (months)**			0.05
1 to 3 months	6.57	1.78	
3 to 6 months	7.81	1.37	
6 to 12 months	7.65	1.42	
>12 months	7.82	1.30	
**Used mobile/tablet**			<**0.001**
No	8.34	1.18	
Yes	7.70	1.37	
**Used computer**			<**0.001**
No	7.62	1.40	
Yes	7.96	1.30	
**Used TV**			0.57
No	7.74	1.37	
Yes	7.58	1.25	
**Screentime with sound/day**			<**0.001**
<2 hours	7.51	1.54	
2 to 6 hours	7.84	1.23	
6 to 12 hours	7.55	1.65	
>12 hours	7.70	1.53	
**Break pattern**			**0.007**
Use with enough break (15 min break after every 2 hours of use)	7.90	1.35	
Use with a small break (10 min break after every 2 hours of use)	7.75	1.28	
Continuous use (4 hours without break)	7.56	1.52	
**Online Entertainment Related Variables**			
**Used mobile/tablet**			0.65
No	7.67	1.40	
Yes	7.74	1.37	
**Used computer**			0.26
No	7.71	1.40	
Yes	7.82	1.29	
**Used TV**			0.07
No	7.69	1.43	
Yes	7.86	1.20	
**Screentime with sound/day**			0.14
<2 hours	7.77	1.39	
2 to 6 hours	7.73	1.31	
6 to 12 hours	7.69	1.44	
>12 hours	7.63	1.70	
**Break pattern**			0.89
Use with enough break (15 min break after every 2 hours of use)	7.89	1.38	
Use with a small break (10 min break after every 2 hours of use)	7.58	1.35	
Continuous use (4 hours without break)	7.71	1.35	

BDT, Bangladeshi Taka (1 USD = 120 BDT)

Table 3 presents the results from multiple linear regression to investigate the factors associated with the SSQ score. The results showed that, after adjusting for all other variables, gender was significantly related to the SSQ score. The SSQ score was 0.295 units lower in females than males (β = −0.295, 95% CI: −0.486 to −0.104, p = 0.002). Individuals without a family history of obesity exhibited higher SSQ scores of 0.264 units than those with such a history (β = −0.264, 95% CI: −0.492 to −0.037, p = 0.023). In terms of duration of use, compared to 1–3 months, engaging in online education for 3–6 months was strongly associated with a 1.338-unit increase in SSQ score (β = 1.338, 95% CI: 0.722 to 1.954, p = 0.000). Similarly, durations of 6–12 months and over 12 months were associated with SSQ score increase of 0.979 units (β = 0.979, 95% CI: 0.427 to 1.530, p = 0.001) and 1.281 units (β = 1.281, 95% CI: 0.744 to 1.819, p = 0.000) respectively. Conversely, using mobile or tablet devices for online education corresponded to lower SSQ scores of 0.818 units compared to not using such devices (β = −0.818, 95% CI: −1.305 to −0.330, p = 0.001). In terms of break patterns, compared to using devices with sufficient breaks (2 hours break after every 2 hours of use), a continuous usage pattern for 4 hours without breaks was associated with a decrease in SSQ score by 0.307 units (β = −0.307, 95% CI: −0.577 to −0.036, p = 0.026). For entertainment purposes, using mobile or tablet devices resulted in higher SSQ scores of 0.448 units higher than not using them (β = 0.448, 95% CI: 0.051 to 0.846, p = 0.027), whereas using a TV resulted in SSQ scores that were 0.185 units higher than not using it (β = 0.185, 95% CI: 0.002 to 0.370, p = 0.049). However, break patterns during online entertainment, compared to using devices with sufficient breaks (2 hours break after every 2 hours of use), taking small breaks (1 hour break after every 2 hours of use) breaks was associated with a decrease in SSQ score by 0.336 units (β = −0.336, 95% CI: −0.568 to −0.104, p = 0.005). Subscale analyses of SSQ has been included in S1 Table.

**Table 3:** Multiple linear regression results for the predictors of SSQ score.

Variables	Coefficient	p-value	Lower bound of 95% CI	Upper bound of 95% CI
**Gender**				
Male	Ref			
Female	−0.295	**0.002**	−0.486	−0.104
**Family history of obesity**				
Yes	Ref			
No	−0.264	**0.023**	−0.492	−0.037
**Online Education-Related Variables**				
**Duration (months)**				
1–3	Ref			
3–6	1.338	**0.000**	0.722	1.954
6–12	0.979	**0.001**	0.427	1.530
>12	1.281	**0.000**	0.744	1.819
**Using mobile or tablet**				
No	Ref			
Yes	−0.818	**0.001**	−1.305	−0.330
**Break Pattern**				
Use with enough break (2 hours break after every 2 hours of use)	Ref			
Use with a small break (1 hour break after every 2 hours of use)	−0.101	0.402	−0.336	0.135
Continuous use (4 hours without break)	−0.307	**0.026**	−0.577	−0.036
**Online Entertainment Related Variables**				
**Using mobile or tablet**				
No	Ref			
Yes	0.448	**0.027**	0.051	0.846
**Using TV**				
No	Ref			
Yes	0.185	**0.049**	0.002	0.370
**Break pattern**				
Use with enough break (2 hours break after every 2 hours of use)	Ref			
Use with a small break (1 hour break after every 2 hours of use)	−0.336	**0.005**	−0.568	−0.104
Continuous use (4 hours without break)	−0.158	0.319	−0.469	0.153

Adjusted for *age*, *education screentime* and *entertainment screentime*

Statistically significant p-values are in bold

CI, Confidence interval

## Discussion

The unprecedented rise in the adoption of online educational platforms has unveiled many concerns, encompassing the potential consequences on the auditory well-being of students. Although noise-induced hearing loss related to personal listening devices and recreational audio exposure has been widely documented, far less attention has been paid to online education as a structured and compulsory listening context. The current study utilized a structured questionnaire, suggesting a potential link between the rapid rise of online education and hearing impairments. During the pandemic, digital learning became a daily requirement, resulting in a significant increase in the usage of digital devices among students. The world is currently witnessing a surge in online education, which has led to a further increase in digital device usage. The current research, the first in the region, examines the possible factors associated with hearing among students continuing online education.

Gender differences showed significant variation, with females showing more susceptibility to poor hearing. Similar findings were also reported by another study in which women reported more subjective hearing problems attributable to headphone usage than men. [18] The underlying reason could be genetic, hormonal [19] or environmental. Additionally, better hearing was reported among students from high socioeconomic status, which may be attributable to better awareness, a more favorable ambient acoustic environment, and the use of higher-quality devices [20].

Individuals with a family history of obesity were more prone to experiencing hearing issues, aligning with prior research indicating an increased association of hearing loss with conditions such as obesity [21], and insomnia [22]. This suggests that a positive family history or genetic predisposition consistently correlates with a higher likelihood of hearing complications.

Headphone users experienced a lower mean SSQ score (7.70) compared to the non-users (7.80). Dirks et al. and Denk et al. have documented a “missing 6 dB” phenomenon, where sound pressure levels required in headphones to achieve equal perceived loudness are significantly higher than sound levels needed in free-field loudspeaker presentations, often ranging from approximately 3 dB to 10 dB higher depending on frequency, acoustical environment, and individual factors [23,24]. This increased exposure level corresponds to a potential elevated risk of auditory damage during prolonged headphone use [25,26].

The respondents with shorter experiences of online education had worse hearing experiences than those with longer durations. This finding mirrors previous studies that identified a relationship between the duration of device use and hearing impairment. [27–29]. According to the studies, the explanation could be increasing participants’ awareness of health issues and their physiological adaptation over time. Specifically, this pattern may reflect an initial period of increased auditory strain and maladaptation during early sustained online learning (3–6 months), partial behavioral or perceptual adaptation over the mid-term (6–12 months), and cumulative or chronic effects of prolonged headphone use and sustained screen-based learning with longer exposure (>12 months). However, longitudinal studies are needed to provide a definitive answer to this question.

Furthermore, this study showed that using portable handheld devices, such as mobile phones and tablets, had a greater impact on respondents’ hearing impairments than using larger-sized devices, such as TVs. The distance between the user and the devices could be the reason behind this [30]. However, this study lacks data on the measurement of distances between students using each of the audio devices. Despite lacking data on individual noise exposure, it can be said that hand-held devices are often used close to the users and are frequently used with hearing equipment (e.g., headphones), which can have a more adverse effect on hearing than other media.

Continuous exposure to sound can irritate the hair cells in the inner ear, leading to sustained damage to hearing. This occurs because prolonged acoustic stimulation can induce metabolic overload, excessive neurotransmitter release at the inner hair cell synapse, and accumulation of reactive oxygen species, all of which contribute to temporary or permanent cochlear and synaptic injury [31]. Online education involves prolonged and continuous exposure to sounds, which can lead to hearing impairment. This current study found a declining trend in hearing among participants who were continuously using gadgets for online education. This finding is consistent with another study that found prolonged headphone use disrupted the high-frequency threshold and significantly impaired balance in adolescents who spent more than eight hours a day in front of a screen [32]. Therefore, ensuring sufficient breaks from using the gadgets can be helpful as a preventive strategy.

The findings were notably different when it came to entertainment purposes, with smartphone and tablet use linked to less hearing damage compared to educational use. This could be explained by the fact that respondents were expected to be more focused on listening to online classes [33], possibly with the highest volume for educational purposes [34]. For entertainment purposes, the use could be more intermittent and relaxed, and perhaps optimal volume could be used as needed, which might explain why the same type of devices caused two opposite impacts on the hearing of respondents, depending on the individual’s purpose. Furthermore, the optimum distance from devices with external audio devices plays a crucial role in auditory health. This study showed that TV played a less harmful role in hearing health when used for entertainment because of the distance from respondents and the delivery of efficient sound frequencies. In contrast, entertainment use with frequent short breaks may involve repeated re-exposure and volume readjustments that fail to allow adequate auditory recovery, potentially producing greater strain than more stable, continuous listening patterns [35]. Taken together, these findings highlight that hearing-related outcomes are strongly context-dependent, influenced by how, why, and under what conditions digital devices are used, rather than solely by device type.

A limitation of this study was the use of a non-probability sampling approach due to limited resources, which may limit the generalizability of findings. However, we included a large sample (almost double the required sample of 422) from across the country, from diverse backgrounds, to address this issue. Although specialist assessment of hearing is the most standard method for assessing hearing, we used a validated tool (sensitivity 85.7% and specificity 86.1%) to assess hearing, which has been used in many previous studies. Causality cannot be established due to the cross-sectional nature of the study design. The questionnaire did not capture specific listening distances or device output levels, which may influence auditory outcomes; future studies should incorporate these measures for a more precise assessment. Despite its limitations, this study is the first to provide valuable insights into hearing among students pursuing online education in Bangladesh, utilizing a large sample size. The novel character of our study is further highlighted by the lack of a thorough body of previous research, which calls for more investigation to validate and build upon our results.

## Conclusion

This current study revealed that online education was related to hearing impairment among students due to prolonged and continuous exposure to noise. Given the inescapable rise in screen usage among students, it is worth considering increasing efforts to educate students about the appropriate use of electronic devices in order to minimize the adverse effects.

## Supporting information

S1 File Questionnaire. (DOCX)

S1 Table Multiple regression results for the subscales of SSQ-12. (DOCX)

S1 File Dataset. (XLSX)
