# Supplementary material for: Online education and its relation to hearing status among higher-secondary students in Bangladesh: A cross-sectional survey
Source: PLoS One. 2026 Feb 13;21(2):e0342668. doi: 10.1371/journal.pone.0342668 (PMC12904458; doi:10.1371/journal.pone.0342668)
Supplement: S1 File — (DOCX) [file pone.0342668.s001.docx]

**Questionnaire**

| ***Background Information (Question 1 – 13)*** |
| --- |
| 1. **Age (in years) *** _______________ 2. **Gender *** ☐ Male ☐ Female ☐ Other ………………. 3. **Educational status *** ☐ SSC ☐ HSC ☐ Undergraduate ☐ Graduate ☐ Post-graduate 4. **Income (in BDT) *** ☐ <10,000 ☐ 10,000 to 30,000 ☐ 30,000 to 60,000 ☐ 60,000 to 1,00,000 ☐ >1,00,000 5. **Does anyone in your family has any of the following? *** ☐ Obesity ☐ Headache ☐ Eye Problem ☐ Insomnia ☐ Hearing Problem ☐ None of the Above 6. **Do you have any of the following? *** ☐ Obesity ☐ Headache ☐ Eye Problem ☐ Insomnia ☐ None of the Above 7. **For how long have you been using digital screen for education? ***   ☐ 1-3 months  ☐ 3-6 months  ☐ 6-12 months  ☐ >12 months   1. **What devices do you use for education**? *****   ☐ Smartphone/tablet  ☐ Computer  ☐ TV   1. **Please mention our daily usage for education. ***   ☐ <2 hours  ☐ 2-6 hours  ☐ 6-12 hours  ☐ >12 hours   1. **Please mention your use pattern for education. ***   ☐ Use with enough break (15 mins break after 2 hours of use)  ☐ Use with small break (10 mins break after 2 hours of use)  ☐ Use without break (≥4 hours without a break)   1. **What devices do you use for entertainment**? *****   ☐ Smartphone/tablet  ☐ Computer  ☐ TV   1. **Please mention our daily usage for entertainment. ***   ☐ <2 hours  ☐ 2-6 hours  ☐ 6-12 hours  ☐ >12 hours   1. **Please mention your use pattern for entertainment. ***   ☐ Use with enough break (15 mins break after 2 hours of use)  ☐ Use with small break (10 mins break after 2 hours of use)  ☐ Use without break (≥4 hours without a break) |

| ***Please answer the following questions regarding your hearing (Question 14 – 25)*** |
| --- |
| Answer on a scale of 0 to 10 points. You may choose any point on the scale, bearing in mind that 0 (on the far left of the scale) means a lot of difficulty performing the activity/situation mentioned in the question, and 10 (on the far right of the scale) means no difficulty performing the activity/situation in the question”   1. You are talking with one other person and there is a TV on in the same room. Without turning the TV down, can you follow what the person you're talking to says? *****   *Not at all*  0    1    2    3    4    5    6    7    8    9    10    *Perfectly*  *Not applicable*   1. You are listening to someone talking to you, while at the same time trying to follow the news on TV. Can you follow what both people are saying? *****   *Not at all*  0    1    2    3    4    5    6    7    8    9    10    *Perfectly*  *Not applicable*   1. You are in conversation with one person in a room where there are many other people talking. Can you follow what the person you are talking to is saying? *****   *Not at all*  0    1    2    3    4    5    6    7    8    9    10    *Perfectly*  *Not applicable*  You are in a group of about five people in a busy restaurant. You can see everyone else in the group. Can you follow the conversation? *****  *Not at all*  0    1    2    3    4    5    6    7    8    9    10    *Perfectly*  *Not applicable*   1. You are with a group and the conversation switches from one person to another. Can you easily follow the conversation without missing the start of what each new speaker is saying? *****   *Not at all*  0    1    2    3    4    5    6    7    8    9    10    *Perfectly*  *Not applicable*   1. You are outside. A dog barks loudly. Can you tell immediately where it is, without having to look? *****   *Not at all*  0    1    2    3    4    5    6    7    8    9    10    *Perfectly*  *Not applicable*   1. Can you tell how far away a bus or a truck is, from the sound? *****   *Not at all*  0    1    2    3    4    5    6    7    8    9    10    *Perfectly*  *Not applicable*   1. Can you tell from the sound whether a bus or truck is coming towards you or going away? *****   *Not at all*  0    1    2    3    4    5    6    7    8    9    10    *Perfectly*  *Not applicable*   1. When you hear more than one sound at a time, do you have the impression that it seems like a single jumbled sound? *****   *Not at all*  0    1    2    3    4    5    6    7    8    9    10    *Perfectly*  *Not applicable*   1. When you listen to music, can you make out which instruments are playing? *****   *Not at all*  0    1    2    3    4    5    6    7    8    9    10    *Perfectly*  *Not applicable*   1. Do everyday sounds that you can hear easily seem clear to you (not blurred)? *****   *Not at all*  0    1    2    3    4    5    6    7    8    9    10    *Perfectly*  *Not applicable*   1. Do you have to concentrate very much when listening to someone or something? *****   *Not at all*  0    1    2    3    4    5    6    7    8    9    10    *Perfectly*  *Not applicable* |
