# Supplementary material for: Online education and its relation to hearing status among higher-secondary students in Bangladesh: A cross-sectional survey
Source: PLoS One. 2026 Feb 13;21(2):e0342668. doi: 10.1371/journal.pone.0342668 (PMC12904458; doi:10.1371/journal.pone.0342668)
Supplement: S1 Table — (DOCX) [file pone.0342668.s002.docx]

**S1 Table: Multiple regression results for the subscales of SSQ-12**

| **Variables** | **SSQ-12 Subscales** | | | | | | | | |
| --- | --- | --- | --- | --- | --- | --- | --- | --- | --- |
|  | **Speech Subscale** | | | **Spatial Subscale** | | | **Qualities of Hearing Subscale** | | |
|  | Coefficient | 95% CI | p-value | Coefficient | 95% CI | p-value | Coefficient | 95% CI | p-value |
| **Gender** |  |  |  |  |  |  |  |  |  |
| Male | - | - | - | Ref. | - | - | - | - | - |
| Female | - | - | - | -0.573 | -0.801, -0.344 | <0.001 | - | - | - |
| **Education Level** |  |  |  |  |  |  |  |  |  |
| SSC | - | - | - | Ref. | - | - | - | - | - |
| HSC | - | - | - | 0.435 | -0.276, 1.146 | 0.23 | - | - | - |
| Undergraduate | - | - | - | 0.759 | 0.026, 1.493 | 0.042 | - | - | - |
| Postgraduate | - | - | - | 1.11 | 0.315, 1.906 | 0.006 | - | - | - |
| **Family history of diseases** |  |  |  |  |  |  |  |  |  |
| Headache | -0.413 | -0.592, -0.234 | <0.001 | -0.325 | -0.544, -0.107 | 0.004 | -0.504 | -0.676, -0.332 | <0.001 |
| Obesity | - | - | - | - | - | - | -0.245 | -0.451, -0.039 | 0.02 |
| **Personal history of diseases** |  |  |  |  |  |  |  |  |  |
| Psychological Problem | -0.901 | -1.219, -0.583 | <0.001 | -0.664 | -1.046, -0.281 | 0.001 | -0.567 | -0.872, -0.261 | <0.001 |
| Obesity | - | - | - | -0.463 | -0.787, -0.14 | 0.005 | - | - | - |
| Eye Problem | - | - | - | - | - | - | 0.225 | 0.053, 0.397 | 0.011 |
| **Headphone Use** | - | - | - | -0.36 | -0.587, -0.134 | 0.002 | - | - | - |
| ***Online education related use*** |  |  |  |  |  |  |  |  |  |
| **Mobile/Tablet Use** | -0.757 | -1.173, -0.342 | <0.001 | - | - | - | - | - | - |
| **Computer Use** | 0.274 | 0.077, 0.471 | 0.006 | 0.321 | 0.093, 0.550 | 0.006 | - | - | - |
| **Duration** |  |  |  |  |  |  |  |  |  |
| 1 to 3 months | Ref. | - | - | Ref. | - | - | Ref. | - | - |
| 3 to 6 months | 0.875 | 0.250, 1.501 | 0.006 | 1.465 | 0.704, 2.226 | <0.001 | 1.625 | 1.032, 2.218 | <0.001 |
| 6 to 12 months | 0.848 | 0.277, 1.419 | 0.004 | 1.091 | 0.405, 1.776 | 0.002 | 1.196 | 0.656, 1.736 | <0.001 |
| >12 months | 1.088 | 0.528, 1.649 | <0.001 | 1.229 | 0.559, 1.899 | <0.001 | 1.448 | 0.920, 1.976 | <0.001 |
| **Screentime with sound/day** |  |  |  |  |  |  |  |  |  |
| <2 hours | Ref. | - | - | - | - | - | Ref. | - | - |
| 2-6 hours | 0.392 | 0.100, 0.683 | 0.008 | - | - | - | 0.278 | 0.002, 0.554 | 0.048 |
| 6-12 hours | 0.07 | -0.284, 0.424 | 0.698 | - | - | - | 0.12 | -0.216, 0.455 | 0.484 |
| >12 hours | 0.442 | -0.107, 0.991 | 0.115 | - | - | - | 0.103 | -0.405, 0.612 | 0.69 |
| **Break pattern** |  |  |  |  |  |  |  |  |  |
| 2 hours break after 2 hours use | Ref. | - | - | - | - | - | Ref. | - | - |
| 1 hours break after 2 hours use | -0.119 | -0.340, 0.102 | 0.292 | - | - | - | -0.087 | -0.295, 0.121 | 0.414 |
| Use without break | -0.41 | -0.67, -0.151 | 0.002 | - | - | - | -0.335 | -0.58, -0.09 | 0.007 |
| ***Online entertainment related use*** | |  |  |  |  |  |  |  |  |
| **Screentime with sound/day** |  |  |  |  |  |  |  |  |  |
| <2 hours | Ref. | - | - | Ref. | - | - | - | - | - |
| 2-6 hours | 0.144 | -0.061, 0.350 | 0.168 | -0.051 | -0.291, 0.189 | 0.675 | - | - | - |
| 6-12 hours | 0.449 | 0.111, 0.788 | 0.009 | -0.375 | -0.735, -0.014 | 0.042 | - | - | - |
| >12 hours | 0.015 | -0.563, 0.593 | 0.959 | -0.039 | -0.687, 0.609 | 0.906 | - | - | - |
| **Break pattern** |  |  |  |  |  |  |  |  |  |
| 2 hours break after 2 hours use | Ref. | - | - | - | - | - | Ref. | - | - |
| 1 hours break after 2 hours use | -0.37 | -0.587, -0.153 | 0.001 | - | - | - | -0.202 | -0.395, -0.008 | 0.041 |
| Use without break | -0.073 | -0.370, 0.224 | 0.629 | - | - | - | 0.011 | -0.236, 0.258 | 0.93 |
| **TV Use** | - | - | - | 0.247 | 0.006, 0.489 | 0.045 | - | - | - |

*Adjusted for age
